# Accurate classification of wheat freeze injury severity from the color information in digital canopy images

**DOI:** 10.1371/journal.pone.0306649

**Published:** 2024-08-09

**Authors:** Jibo Zhang, Haijun Huan, Can Qiu, Qi Chen, Chuanxiang Yi, Pei Zhang

**Affiliations:** 1 Key Laboratory for Meteorological Disaster Prevention and Mitigation of Shandong, Jinan, China; 2 Shandong Climate Center, Jinan, China; 3 Zibo Meteorological Bureau, Zibo, China; 4 Key Laboratory of Crop Physiology and Ecology in Southern China, Ministry of Agriculture, Jiangsu Collaborative Innovation Center for Modern Crop Production, National Engineering and Technology Center for Information Agriculture, Nanjing Agricultural University, Nanjing, China; 5 Jiangsu Meteorological Bureau, Nanjing, China; Nuclear Science and Technology Research Institute, ISLAMIC REPUBLIC OF IRAN

## Abstract

This paper explores whether it is feasible to use the RGB color information in images of wheat canopies that were exposed to low temperatures during the growth season to achieve fast, non-destructive, and accurate determination of the severity of any freeze injury it may have incurred. For the study presented in this paper, we compared the accuracy of a number of algorithmic classification models using either meteorological data reported by weather services or the color gradation skewness-distribution from high-definition digital canopy images acquired in situ as inputs against a reference obtained by manually assessing the severity of the freeze injury inflicted upon wheat populations at three experimental stations in Shandong, China. The algorithms we used to construct the models included in our study were based on either K-means clustering, systematic clustering, or naïve Bayesian classification. When analyzing the reliability of our models, we found that, at more than 85%, the accuracy of the Bayesian model, which used the color information as inputs and involved the use of prior data in the form of the reference data we had obtained through manual classification, was significantly higher than that of the models based on systematic or the K-means clustering, which did not involve the use of prior data. It was interesting to note that the determination accuracy of algorithms using meteorological factors as inputs was significantly lower than that of those using color information. We also noted that the determination accuracy of the Bayesian model had some potential for optimization, which prompted us to subject the inputs of the model to a factor analysis in order to identify the key independent leaf color distribution parameters characterizing wheat freeze injury severity. This optimization allowed us to improve the determination accuracy of the model to over 90%, even in environments comprising several different ecological zones, as was the case at one of our experimental sites. In conclusion, our naïve Bayesian classification algorithm, which uses six key color gradation skewness-distribution parameters as inputs and involves the use of prior data in the form of manual assessments, qualifies as a contender for the development of commercial-grade wheat freeze injury severity monitoring systems supporting post-freeze management measures aimed at ensuring food security.

## 1. Introduction

Safeguarding food security is an essential requirement for maintaining global peace and stability [1, 2], but the increasingly frequent and severe consequences of extreme weather events and climate change brought about by global warming make fulfilling this task more and more arduous [3–5]. Wheat, as one of the world’s most widely grown and highest-yielding food crops [6], plays an important role in guaranteeing global food security. Overwinter freeze injury to winter wheat crops is common worldwide and in severe cases, it can result in a 30–50% yield reduction [7]. In the wheat growing areas in northern China, freeze injury is considered to be the biggest meteorological disaster befalling the farming community [8]. During our experiment, which ran from November 2022 to January 2023, the region where several of the experimental sites included in our study were located was subjected to a severe cold spell, confirming the current trend towards low-temperature spells becoming more frequent and more severe. This climatic development can be expected to exacerbate the negative impacts of freeze injury in times to come, thereby jeopardizing the stability of winter wheat production systems and increasing the risk of winter wheat yield losses [9]. Evidently, in order to ensure food security, it is imperative to timely and accurate monitor and assess the impact of freeze injury on winter wheat production systems so that appropriate remedial measures can be taken as early as possible.

Traditionally, the severity and extent of freeze injury incurred by wheat crops was usually assessed through field surveys conducted by agricultural technicians [10]. Surveys, however, have numerous limitations: they are affected by changes in the ambient environmental conditions (wind, sun angle, temperature, humidity) and circadian rhythms [11] and often suffer from inefficiencies and difficulties with respect to data quality [12]. As the development of science and technology progresses, more and more researchers looking to provide an early warning system for wheat yield losses from freeze injury have turned to utilizing multi-source information such as satellite remote sensing data and hyperspectral imagery [13–19]. Several researchers have reported successfully utilizing remote sensing image data from optical remote sensing satellites (for example optical satellites HJ-1 A and B), MODIS satellites, and multi-temporal GF-1 satellites for monitoring the severity of winter wheat freeze injury [13, 20, 21]. In addition, Feng et al. (2018) [22] found that the hyperspectral reflectance of wheat canopies at the seedling stage was significantly affected by freeze injury, allowing them to ascertain the best period and simulation techniques for estimating yield losses due to winter wheat freezing damage from hyperspectral images. However, satellite remote sensing and hyperspectral imaging techniques exhibit several shortcomings; they tend to be associated with a relatively low spatial and temporal resolution and a relatively high cost [23, 24], making them unsuitable for most agricultural meteorological services. Recently, however, the increasing availability of affordable high-resolution digital camera equipment has made the deployment of digital imagery much more accessible, and, as a result, a growing number of studies have started to utilize digital images for both qualitative and quantitative characterization of the phenotypic traits of crop plants [25, 26]. Reported findings include that the color of leaves gleaned from a digital image can be used as an indicator for crop growth and environmental stress [27–30]. Elaborating on these reports, Chen et al. (2020) [31] proposed a method for deriving a number of crop damage indicators from the so-called Color Gradation Skewness-Distribution (CGSD) parameters extracted from the leaf color information in digital canopy images, which heralded the use of digital images for rapid and nondestructive assessment of wheat freeze injury severity [32]. In an attempt to further Chen’s proposal, the study presented in this paper outlines a naïve Bayesian algorithm that takes a number of these CGSD parameters as inputs in order to provide a classification for the severity of any freeze injury it has incurred. It should be noted that, when applied to image classification, models based on Bayesian classification algorithms tend to be more successful when using prior data to reduce overfitting during training; using a prior improves the algorithm’s robustness and capacity for making generalizations.

Our study used high-definition digital images of wheat canopies made before and after the winter wheat growing season by crop growth observers in wheat fields at research stations at the following three locations in Shandong Province: Gaomi City (resorting under Weifang City), Jiyang District (resorting under Jinan City) and Qihe County (resorting under Dezhou City). By combining a prior obtained through manual classification with data analyses based on K-means clustering, systematic clustering and naïve Bayesian classification, we managed to construct a model capable of rapidly and non-destructively classifying wheat freeze injury severity from the leaf color information in digital wheat canopy images. We hope that our study can improve the accuracy and timeliness of the freeze injury monitoring, provide technological support for mitigating the impacts of freeze injury and help limiting yield losses due to cold weather.

## 2. Materials and methods

### 2.1 Experimental design

**Fig 1:**
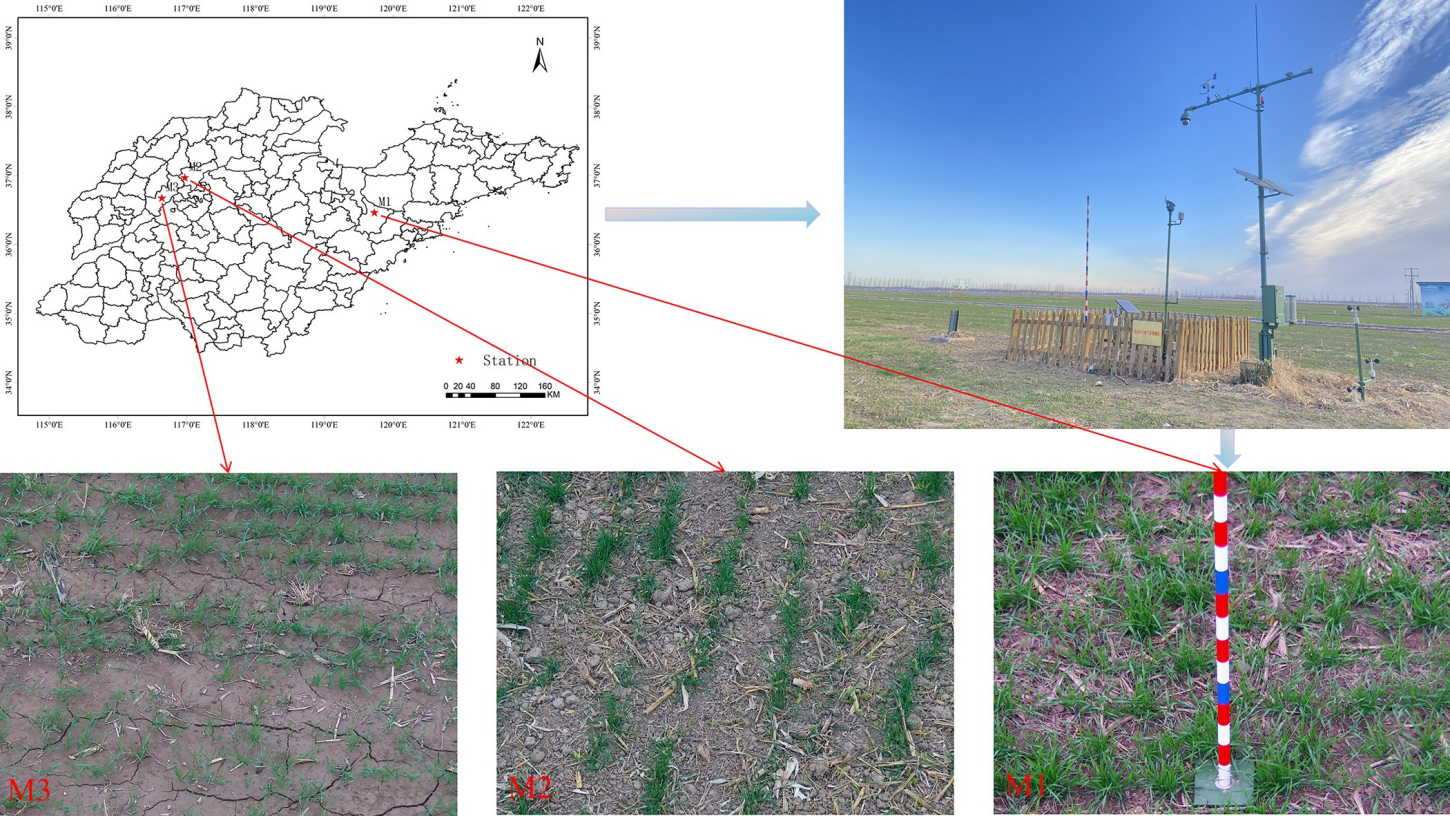
Geographical location of the three experimental sites in Shandong Province, China, along with photographs showing the camera mounting installation as well as the environmental conditions at each site.

Our study involves data from three experimental sites. One of these was located in Jiangzhuang Town, which resorts under Gaomi City, Weifang City in eastern Shandong Province (36°28’12"N, 119°43’48"E). The other two were at the Experimental Station of the Shandong Academy of Agricultural Sciences in Taiping Town, which is in the Jiyang District of Jinan City (36°58’38"N, 116°58’282"E), and in Jiaomiao Town in Qihe county, which resorts under Dezhou City (N36°40’32"N, 116°38’56"E), both in northern Shandong Province (see Fig 1).

As the experimental subjects we used the wheat variety Yangguang 818 at the Gaomi site, Jimai 44 at the Jiyang site and Jimai 22 at the Qihe site.

The experimental wheat was sowed using strip sowing with a row spacing of 0.2 m. It should be noted that local differences between the growing environments in the northern and eastern regions of Shandong affected the start of the wheat growing season, which is why the sowing date we used for the Gaomi site was earlier than that used for the other two sites.

### 2.2 Collection of canopy images

The wheat canopy images we used for our study were acquired in situ, using a camera with an image resolution of 1920×1080 pixels (model number: DH-SD-65F630U-HN-Q; manufacturer: Zhejiang Dahua Technology Co. Ltd, China), which was mounted on top of a mast measuring 580 cm in height (see Fig 1). When creating the images, we applied a fixed focal length exposure and the automatic white balance feature provided by the camera. Images were acquired every day at 16:00 for the duration of the experiment. At the Gaomi site, the experiment ran from November 22, 2022 to January 21, 2023; at the Jiyang site it ran from November 10, 2022 to January 13, 2023, and at the Qihe site it ran from November 23, 2022 to January 26, 2023. To improve the accuracy of the color gradation, we discarded images acquired during conditions of intense lighting, frost or snow cover, leaving us with a total of 78 canopy images we deemed usable as input for our analysis; 16 images were acquired at the Gaomi site, 31 at the Jiyang site, and 31 at the Qihe site (see Table 2).

### 2.3 Meteorological data acquisition

Our experiment used meteorological data collected by in-situ microclimate observatories at each site, as well as meteorological data obtained from the national weather stations in Gaomi, Jiyang and Qihe. The Shandong Meteorological Information Center codes representing the meteorological factors used in our study are shown in Table 1.

**Table 1:** Meteorological factors used in our experiment.

Meteorological factor code	Description
*Z*1	Effective accumulated temperature since sowing
*Z*2	Daily mean temperature
*Z*3	Daily mean temperature over 3 days moving
*Z*4	Daily mean temperature over 5 days moving
*Z*5	Daily mean temperature at 30 cm in wheat fields
*Z*6	Daily mean temperature at 30 cm over 3 days moving in wheat fields
*Z*7	Daily mean temperature at 30 cm over 5 days moving in wheat fields
*Z*8	Daily mean temperature at 60 cm in wheat fields
*Z*9	Daily mean temperature at 60 cm over 3 days moving in wheat fields
*Z*10	Daily mean temperature at 60 cm over 5 days moving in wheat fields
*Z*11	Daily mean temperature at 150 cm in wheat fields
*Z*12	Daily mean temperature at 150 cm over 3 days moving in wheat fields
*Z*13	Daily mean temperature at 150 cm over 5 days moving in wheat fields
*Z*14	Daily minimum temperature
*Z*15	Daily minimum temperature over 3 days moving
*Z*16	Daily minimum temperature over 5 days moving
*Z*17	24-hour temperature change
*Z*18	24-hour temperature change over 3 days moving
*Z*19	24-hour temperature change over 5 days moving
*Z*20	Daily mean dew point temperature
*Z*21	Daily mean dew point temperature over 3 days moving
*Z*22	Daily mean dew point temperature over 5 days moving
*Z*23	Daily mean grass temperature
*Z*24	Daily mean grass temperature over 3 days moving
*Z*25	Daily mean grass temperature over 5 days moving
*Z*26	Daily mean relative humidity
*Z*27	Daily mean relative humidity over 3 days moving
*Z*28	Daily mean relative humidity over 5 days moving
*Z*29	Daily precipitation
*Z*30	Daily precipitation over 3 days moving
*Z*31	Daily precipitation over 5 days moving

### 2.4 Determination of the CGSD parameters

To determine the CGSD parameters characterizing wheat freeze injury severity, we used the method proposed by Chen et al. (2020) [31], which involves obtaining a histogram of the cumulative color gradation from the canopy images we acquired, and separating, denoising, and ordering the resulting RGB information. Subsequent application of mean, median, mode, skewness, and kurtosis functions yields the following 20 CGSD parameters: *R*_Mean_, *R*_Median_, *R*_Mode_, *R*_Skewness_, *R*_Kurtosis_, *G*_Mean_, *G*_Median_, *G*_Mode_, *G*_Skewness_, *G*_Kurtosis_, *B*_Mean_, *B*_Median_, *B*_Mode_, *B*_Skewness_, *B*_Kurtosis_, *Y*_Mean_, *Y*_Median_, *Y*_Mode_, *Y*_Skewness_ and *Y*_Kurtosis_, where the quantities labeled R refer to the red channel of the image, those labeled G refer to the green channel, those labeled B refer to the blue channel, and those labeled Y refer to the gray-level image. A detailed description of how these parameters were extracted from the images can be found in the aforementioned paper by Chen et al.

### 2.5 Classification of wheat freeze injury severity

#### 2.5.1 Manual classification of wheat freeze injury severity according to the standard

Our study deployed the services of three agrometeorological observers with associate senior titles who performed daily manual assessments of the severity of the wheat freeze injury inflicted upon the experimental crops through on-site inspection of the wheat leaf color and the extent of any frozen leaf areas. Our consultants followed the “Specification for Agrometeorological Observations” [33], which classifies the severity of observed wheat freeze injury into the following three categories: no freeze injury, mild freeze injury, and severe freeze injury. In the subsequent analysis, we assigned 0 to no freeze injury, 1 to mild freeze injury, and 2 to severe freeze injury (see Table 2).

**Table 2:** Number of samples and sample usage for each site.

Degrees of frost damage	Grade assignment	Number of samples (sheets)		
		Modeling group	Verification group	Applied Testing Group
No freeze injury	0	19	57	124
Mild freeze injury	1	16	48	0
Severe freeze injury	2	12	36	0
Total		47	141	124

#### 2.5.2 Classification using clustering and Bayesian methods

To classify the severity of wheat freeze injury from the CGSD characteristics extracted from canopy images, we used algorithms involving K-means clustering, systematic clustering and Bayesian classification based upon meteorological factors or CGSD parameters extracted from digital canopy images.

K-means clustering and systematic clustering are commonly used for algorithmic classification methods [34, 35]; neither of these needs a prior, meaning they can both obtain results directly from the input variables, which is typical for this type of unsupervised learning algorithm. Bayesian classification methods, on the other hand, use a so-called naïve Bayesian classifier, which needs to be obtained through training the algorithm with prior data in order to build a probabilistic model. Only after completing the training step, the model can be used to classify new data [36–38].

**Fig 2:**
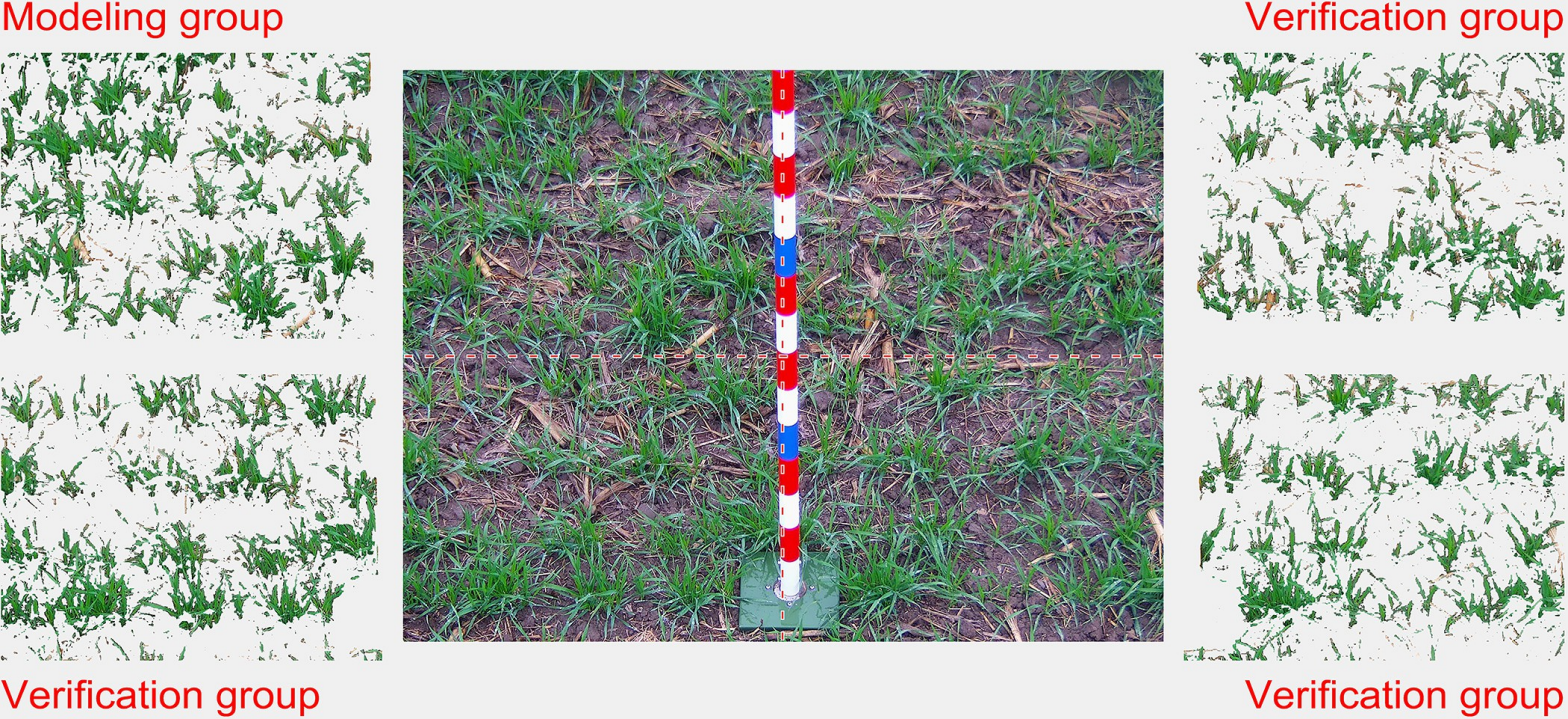
Wheat image grouping.

For our study, we decided to divide the canopy images into four equal sections, and use each section as a sample to be used as input for our algorithms: The upper-left section of each image at Gaomi and Jiyang stations was selected to construct the modeling group sample, with a total of 47 images; the remaining three sections were used for validation, with a total of 141 images (see Fig 2). In addition, the images acquired at the Qihe station were also divided into four equal sections and used for applied testing, with a total of 124 images (see Table 1).

Using either the 20 CGSD parameters extracted from the 47 modeling samples shown in Table 2 or the 31 meteorological factors shown in Table 1 as inputs for models based on one of the aforementioned algorithms, we constructed the wheat freeze injury class classification models shown in Table 3: Models F1 and F2 use meteorological factors and CGSD parameters as inputs for a systematic clustering algorithm, respectively; Models F3 and F4 use meteorological factors and CGSD parameters as inputs for a K-means algorithm, respectively; and Model F5 uses CGSD parameters as inputs for a Bayesian classification model while using the same categories as those used for manual assessment of the wheat freeze injury severity (see section “2.5.1 *Manual Classification of wheat freeze injury severity according to the standard”*) as the output variables.

**Table 3:** Experimental models for classifying wheat freeze injury severity.

Model	Inputs	Modeling algorithm
F1	Meteorological factors	Systematic clustering
F2	CGSD parameters	
F3	Meteorological factors	K-Means clustering
F4	CGSD parameters	
F5	CGSD parameters	Bayesian classification

#### 2.5.3 Detailed description of the factor analysis used to optimize model F5

Factor analyses can be used to extract a limited number of indicators that characterize a large set of variables, thus simplifying the task of screening and structuring the data. Using the factor analysis method proposed by Zhang et al. (2022) [32], we reduced the set of 20 CGSD parameters to a total of six key indicators for wheat freeze injury severity, and used those as inputs for an optimized version of the Bayesian model F5. Our factor analysis included the steps described below.

1. We used the top-left sections of the 47 images acquired at the Gaomi and Jiyang stations to determine the 20 CGSD parameters described in section “2.4 Determination of the CGSD parameters”. The resulting parameters were subjected to the Kaiser-Meyer-Olkin (KMO) test and Bartlett’s sphericity test, and the samples for which the KMO value was higher than 0.7 while the P value associated with Bartlett’s test was lower than 0.05 were qualified to be passed on to the next step of factor analysis.
2. We then determined the most significant composite factor based on the eigenvalues, cumulative contribution ratios, and fragmentation diagrams characterizing each parameter.
3. Using the index *i* to enumerate the CGSD parameters, we calculated a principal component score for each parameter using the following formula:

$$F{SC}_{i}=\sum_{j=1}^{n}{VDR}_{j}\times {CDR}_{j}\times {SC}_{ij}$$ (1)

In this formula, *FSC*_*i*_ denotes the principal component score; *VDR*_*j*_ denotes the variance contribution of composite factor *j; CDR*_*j*_ is the cumulative contribution rate of composite factor *j*; and *SC*_*ij*_ is the contribution of composite factor *j* to parameter *i*. The five parameters with the highest absolute value of their principal component score were identified as the key indicators of wheat freeze injury severity.

2.5.4 Accuracy determination of nominal wheat freeze injury severity classification algorithms

Based on the classification results from the agrometeorological observers in 2.5.1, the wheat freeze injury severity classification accuracy of the classification algorithms in 2.5.2 and 2.5.3 was calculated. The formula is as follows:

$$Classification\;accuracy=\frac{The\;number\;of\;samples\;exactly\;consistent\;with\;the\;agrometeorological\;observers}{Total\;number\;of\;samples}\times 100\%$$ (2)

## 3. Results

### 3.1 Freezing-induced changes in the color distribution of wheat canopy images

**Fig 3:**
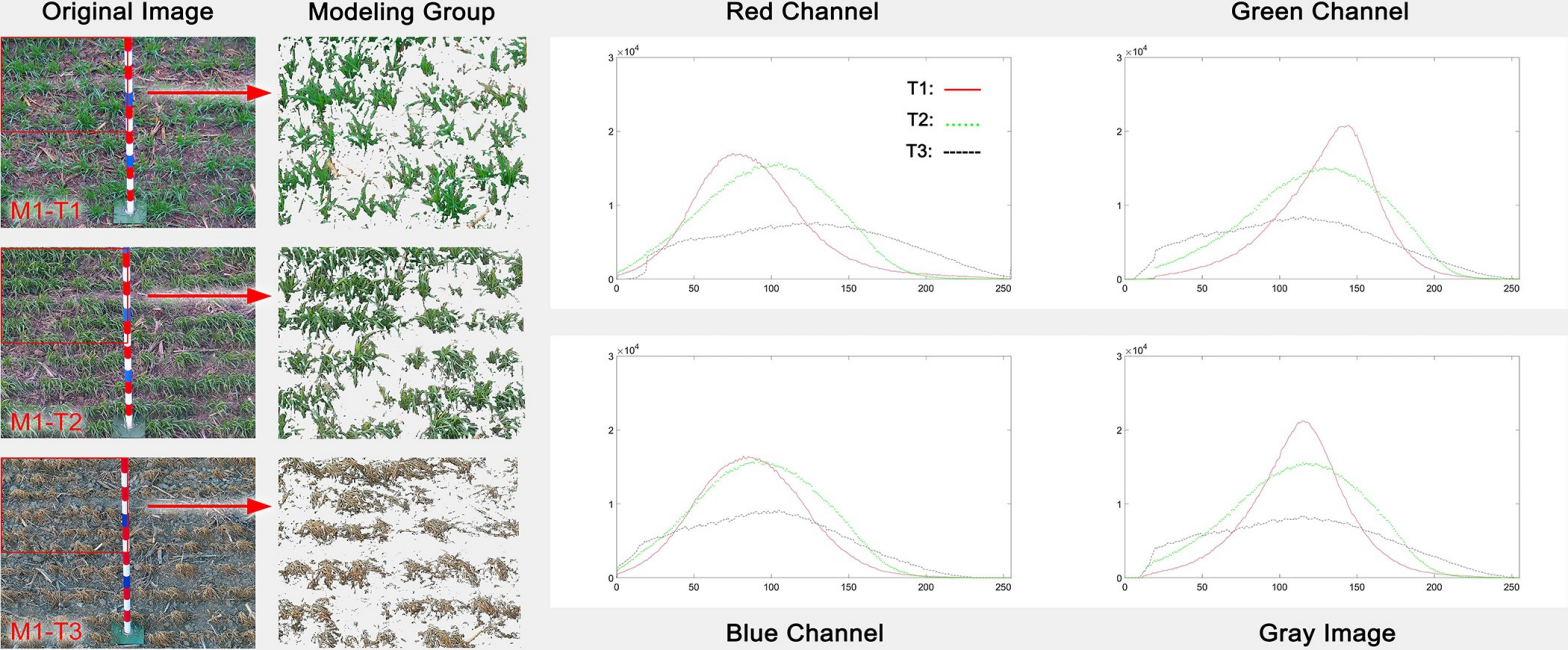
Color histograms for digital canopy images of wheat populations at Gaomi station exhibiting different degrees of freeze injury, broken down by RGB channel.

Plants exposed to severe abiotic stress tend to suffer from significant impairments to their growth and development. The most common adversity experienced by winter crops is freeze injury due to excessive cooling under the influence of cold air during cold weather periods. In order to determine whether it is possible to assess the severity of freeze injury suffered by wheat crops from digital canopy images, we selected three images of the canopies of wheat populations at Gaomi station that, according to the manual assessment described in section “2.5.1 Mannual Classification of wheat freeze injury severity according to the standard”, exhibited different degrees of freeze injury, and subjected them to a comparative analysis with respect to histograms revealing their color scale distribution and color bias (Fig 3). Our findings indicated that the color distribution histograms of images of canopies affected by various degrees of freeze injury exhibited an amount of shifting, flattening and skewing that was directly proportional to the degree of freeze injury. The color shift we observed suggests that the color of a wheat canopy turns lighter after being exposed to freezing; evidently, the leaf color gradually changed from bright green to yellow-green and ultimately to yellow. The flattening and skewing we observed indicates that the number of leaf colors present in the canopy increases with the severity of the freeze injury: in the absence of freeze injury, the canopy leaf color is bright green, which is reflected by its leaf color scale value being relatively concentrated; under the influence of freeze injury, however, the canopy color changes from bright green to yellow-green, thereby introducing additional colors that spread out the leaf color histogram and flatten and skew its shape. The color of leaves that were afflicted by severe freeze injury was a neutral yellow, which confirms that changes in the distribution of leaf colors in a wheat canopy are proportional to the severity of the freeze injury it suffered.

### 3.2 Wheat freeze injury severity correlation with meteorological factors and CGSD parameters

The wheat freeze injury severity exhibited a significantly correlation with the meteorological factors included in our study (Table 4). At p < 0.01, Z1 was significantly positively correlated with the wheat freeze injury severity while Z2-16 and Z20-28 were significantly negatively correlated. It should be noted that except for cumulative temperature, the farmland microclimate factors (Z5-13) exhibited a stronger correlation with the freeze injury severity than the other meteorological factors, as reflected in the absolute values of the corresponding correlation coefficients exceeding 0.7.

**Table 4:** Correlations between meteorological factors and the observed freeze injury severity.

Meteorological factor	Correlation coefficient	Meteorological factor	Correlation coefficient	Meteorological factor	Correlation coefficient
**Z1**	0.820**	**Z11**	-0.636**	**Z21**	-0.559**
**Z2**	-0.436**	**Z12**	-0.702**	**Z22**	-0.579**
**Z3**	-0.491**	**Z13**	-0.741**	**Z23**	-0.455**
**Z4**	-0.521**	**Z14**	-0.413**	**Z24**	-.505**
**Z5**	-0.700**	**Z15**	-0.455**	**Z25**	-0.534**
**Z6**	-0.753**	**Z16**	-0.463**	**Z26**	-0.480**
**Z7**	-0.781**	**Z17**	0.239	**Z27**	-0.587**
**Z8**	-0.677**	**Z18**	0.242	**Z28**	-0.674**
**Z9**	-0.743**	**Z19**	0.043	**Z29**	-0.140
**Z10**	-0.780**	**Z20**	-0.494**	**Z30**	-0.203
				**Z31**	-0.224

A value being annotated with two asterisks (**) indicates it exhibits a significant correlation with the freeze injury severity at p < 0.01.

The wheat freeze injury severity also exhibited a significant correlation with the CGSD parameters extracted from images of an affected wheat canopy (Table 5). At p < 0.01, all five leaf color parameters extracted from the red (R) channel of the leaf color distribution (*R*_Mean_, *R*_Median_, *R*_Mode_, *R*_Skewness_ and *R*_Kurtosis_), as well as the kurtosis parameter extracted from the green (G) channel (*G*_kurtosis_), the mean and median parameters extracted from the blue (B) channel (*B*_Mean_, *B*_Median_) and the mean, median and kurtosis parameters extracted from the gray-level image (*Y*_Mean_, *Y*_Median_ and *Y*_Kurtosis_) were significantly correlated with the observed wheat freeze injury severity, and at p < 0.05, the same was true for the plurality and kurtosis parameters extracted from the B channel (*B*_Mode_ and *B*_Kurtosis_) as well as for the plurality parameter extracted from the gray-level image (*Y*_Mode_). It is interesting to note that although both the CGSD parameters extracted from the leaf color distribution and the meteorological factors exhibited evidence of being strongly correlated with the wheat freeze injury severity, the magnitude and direction of the correlations we observed for the CGSD parameters was varied: The parameters associated with leaf color depth (mean value, median value, and plurality) tended to be strongly and positively correlated with the wheat freeze injury severity, whereas those associated with the color distribution (skewness and kurtosis) tended to be less strongly and negatively correlated.

**Table 5:** Correlations between the CGSD parameters extracted from canopy images and the corresponding observed freeze injury severity.

CGSD parameters	Correlation coefficient	CGSD parameters	Correlation coefficient
*R*_Mean_	0.777**	*B*_Mean_	0.451**
*R*_Median_	0.765**	*B*_Median_	0.409**
*R*_Mode_	0.774**	*B*_Mode_	0.344*
*R*_Skewness_	-0.528**	*B*_Skewness_	-0.150
*R*_Kurtosis_	-0.545**	*B*_Kurtosis_	-0.331*
*G*_Mean_	0.187	*Y*_Mean_	0.514**
*G*_Median_	0.105	*Y*_Median_	0.455**
*G*_Mode_	-0.044	*Y*_Mode_	0.290*
*G*_Skewness_	0.213	*Y*_Skewness_	-0.049
*G*_Kurtosis_	-0.423**	*Y*_Kurtosis_	-0.497**

A value being annotated with one asterisk (*) or two asterisks (**) indicates it exhibits a significant correlation with the freeze injury severity at p < 0.05 or p < 0.01, respectively.

### 3.3 Accuracy of nominal wheat freeze injury severity classification algorithms

To assess the accuracy of the classification algorithms described in section “2.5.2 Classification using clustering and Bayesian methods”, we compared the classifications they provided against the results obtained through manual assessment.

With respect to the clustering algorithms, which do not involve the use of prior data, we found that the accuracy of the classification algorithms based on systematic clustering or K-means clustering using wheat canopy leaf color information as the independent variable (F2 and F4) were 63.83% and 72.34%, respectively, while the accuracy of the corresponding algorithms using meteorological factors as the independent variable (F1 and F3) was only 4.20% and 23.04%, respectively (Table 6). Evidently, classification based on clustering leaf color information is significantly more accurate than classification based on clustering meteorological factors, but nevertheless, even the former only reached an accuracy level of about 70%, which is insufficient for the purpose of commercial wheat freeze injury monitoring.

**Table 6:** Accuracy of wheat freeze injury severity classification algorithms not involving the use of prior data.

Clustering model	F1	F2	F3	F4
**Accurate classifications**	2	30	11	34
**Accuracy rate (n = 47)**	4.20%	63.83%	23.40%	72.34%

Bayesian classification algorithms do involve the use of prior data, and for the naïve Bayesian classification algorithm used for our study (F5) we utilized the manually assessed wheat freeze injury severity classification (see section “2.5.1 Manual Classification of wheat freeze injury severity according to the standard”) to fulfill this purpose. As the independent variable, we used the CGSD parameters extracted from the modeling sections of canopy images acquired at the Gaomi and Jiyang stations. After analyzing the classification results we obtained by subjecting the modeling samples to F5 (Table 7), we found that its accuracy, which ranged from 81.25% to 100% with an average value of 87.23%, was significantly higher than that of the algorithms that do not involve the use of prior data. When using the validation samples, the accuracy ranged from 85.42% to 100%, with an average value of 90.07%.

**Table 7:** Accuracy of the Bayesian classification algorithm used in our study (F5).

Classification after manual assessment	Classification after applying F5 to modeling samples			Accuracy (n = 47)
	No freeze injury	Mild freeze injury	Severe freeze injury	
No freeze injury	16	3	0	84.21%
Mild freeze injury	2	13	1	81.25%
Severe freeze injury	0	0	12	100.00%
	Average accuracy:			87.23%
Classification after manual assessment	Classification after applying F5 to validation samples			Accuracy (n = 141)
	No freeze injury	Mild freeze injury	Severe freeze injury	
No freeze injury	50	5	2	87.72%
Mild freeze injury	4	41	3	85.42%
Severe freeze injury	0	0	36	100.00%
	Average accuracy:			90.07%

### 3.4 Optimization of the Bayesian classification model

In the light of the accuracy analysis provided in the previous section, we concluded that the best approach to monitoring wheat freeze injury severity is to deploy a Bayesian algorithm that involves using prior data. However, analysis of the results obtained by applying the nominal Bayesian algorithm F5 described before to canopy images acquired at Qihe station revealed that the accuracy it achieved in this practical application, which included cross-ecological zones, was only 66.13% (Table 8), and therefore below the commercially required accuracy level. In this section, we propose and evaluate several optimizations of the Bayesian classification model aimed at improving its accuracy in this type of situation.

**Table 8:** Accuracy of the Bayesian classification model F5 when applied to the subset of canopy images acquired at Qihe station.

Classification after manual assessment	Classification after applying F5 to modeling samples			Accuracy (n = 124)
	No freeze injury	Mild freeze injury	Severe freeze injury	
No freeze injury	82	0	42	66.13%
Mild freeze injury	0	0	0	n/a
Severe freeze injury	0	0	0	n/a
	Average accuracy			66.13%

In our attempt to optimize the F5 model, we took a closer look at the 20 CGSD parameters it uses a inputs. Although these parameters can comprehensively characterize the leaf condition, we found evidence of covariance among them, and, as previously reported, the accuracy of a classification model using too many covariate variables as inputs can end up being compromised (Zhang et al. 2020). To overcome this issue, we carried out a factor analysis to determine which subset of the 20 CGSD parameters we extracted were the key indicators for wheat freeze injury, thus allowing us to disregard less relevant covariate parameters and removing any interference they may introduce. Before carrying out our factor analysis, we first subjected the CGSD parameters to a sphericity test. The KMO value we found was 0.781, and the associated P-value according to Bartlett’s test was close to zero, indicating that the data was suitable for being subjected to factor analysis. After performing the sphericity test we carried out an ANOVA analysis on the parameter set, the results of which are shown in Table 9. The results enabled us to identify the two most promising composite factors (labeled “1” and “2” in Table 9), which were characterized by having an eigenvalue significantly greater than one as well as a significant cumulative variance contribution. A loading analysis of these two composite factors, the results of which are shown in Table 10, revealed that the parameters with the highest load with respect to composite factor 1 were *G*_Skewness_, *G*_Median_, *G*_Mode_, *G*_Mean_, *Y*_Skewness_, and Y_Mode_, and that those with the highest load with respect to composite factor 2 were *R*_Kurtosis_, *Y*_Kurtosis_, *G*_Kurtosis_, *R*_Mode_, *R*_Skewness_, and *B*_Kurtosis_. Further analysis of the scores of these parameters allowed us to identify the six key parameters reflecting the bias of the RGB color distribution of the canopy images as *R*_Kurtosis_, *G*_Skewness_, *G*_Median_, *G*_Mode_, *Y*_Skewness_, and *G*_Mean_.

**Table 9:** Analysis of variance (ANOVA) for combined factors.

Composite factor	Eigenvalue	Variance contribution (%)	Cumulative variance contribution (%)
1	14.963	74.815	74.815
2	2.751	13.757	88.572
…	…	…	…
20	2.525E-05	1.262E-04	100.000

**Table 10:** Parameter loadings and scores of edge parameters in the composite factor after rotation of the combined factor.

	Variable loads (after rotation)		Variable scores		Principal component score coefficients		
	Load with respect to composite factor 1	Load with respect to composite factor 2	Score with respect to composite factor 1	Score with respect to composite Factor 2	Score coefficient with respect to composite factor 1	Score coefficient with respect to composite factor 2	Absolute value of the sum of the principal score coefficients
*R*_Mean_	0.503	0.753	-0.009	0.096	-0.008	0.015	0.007
*R*_Median_	0.514	0.755	-0.007	0.095	-0.006	0.015	0.009
*R*_Mode_	0.448	0.789	-0.026	0.113	-0.022	0.018	0.004
*R*_Skewness_	-0.440	-0.786	0.027	-0.113	0.023	-0.018	0.005
*R*_Kurtosis_	0.074	-0.947	0.155	-0.226	0.131	-0.035	**0.096**
*G*_Mean_	0.915	0.342	0.136	-0.058	0.115	-0.009	**0.106**
*G*_Median_	0.948	0.272	0.153	-0.078	0.129	-0.012	**0.117**
*G*_Mode_	0.945	0.164	0.168	-0.103	0.142	-0.016	**0.126**
*G*_Skewness_	-0.959	-0.001	-0.195	0.141	-0.165	0.022	**0.143**
*G*_Kurtosis_	-0.249	-0.885	0.080	-0.164	0.068	-0.025	0.042
*B*_Mean_	0.633	0.701	0.025	0.065	0.021	0.010	0.031
*B*_Median_	0.671	0.673	0.037	0.053	0.031	0.008	0.040
*B*_Mode_	0.641	0.647	0.035	0.052	0.029	0.008	0.038
*B*_Skewness_	-0.802	-0.450	-0.097	0.016	-0.082	0.003	0.079
*B*_Kurtosis_	-0.403	-0.786	0.034	-0.119	0.029	-0.018	0.010
*Y*_Mean_	0.763	0.594	0.067	0.022	0.057	0.003	0.060
*Y*_Median_	0.804	0.561	0.081	0.008	0.068	0.001	0.069
*Y*_Mode_	0.828	0.516	0.092	-0.005	0.078	-0.001	0.077
*Y*_Skewness_	-0.897	-0.320	-0.135	0.060	-0.114	0.009	**0.105**
*Y*_Kurtosis_	-0.145	-0.941	0.110	-0.192	0.093	-0.030	0.063

We then optimized the Bayesian classification model F5 by limiting the input parameters to these six key parameters, thus obtaining model F5_Optimized_. The test results of applying F5_Optimized_ to the acquired samples are shown in Table 11; the results indicate that the overall accuracy of F5_Optimized_ with respect to the modeling samples and the validation samples from the same image is close to that of model F5, and that, at 90.32%, it is significantly better than that of F5 with respect to the validation samples from other images.

**Table 11:** Accuracy of the optimized Bayesian classification model F5_Optimized_.

Classification after manual assessment	Classification after applying F5_Optimized_ to modeling samples			Accuracy (n = 47)
	No freeze injury	Mild freeze injury	Severe freeze injury	
No freeze injury	18	1	0	94.74%
Mild freeze injury	2	14	0	87.50%
Severe freeze injury	1	0	11	91.67%
	Average accuracy			91.49%
Classification after manual assessment	Classification after applying F5 _Optimized_ to validation samples from the same image			Accuracy (n = 141)
	No freeze injury	Mild freeze injury	Severe freeze injury	
No freeze injury	52	1	4	91.23%
Mild freeze injury	8	40	0	83.33%
Severe freeze injury	2	0	34	94.44%
	Average accuracy			89.36%
Classification after manual assessment	Classification after applying F5_Optimized_ to validation samples from other images			Accuracy (n = 124)
	No freeze injury	Mild freeze injury	Severe freeze injury	
No freeze injury	112	2	10	90.32%
Mild freeze injury	0	0	0	n/a
Severe freeze injury	0	0	0	n/a
	Average accuracy			90.32%

## 4. Discussion

Wheat freeze injury frequently befalls winter wheat crops exposed to low temperatures during their growth period. At present, the most common way to monitor the severity of winter wheat freeze injury is to evaluate meteorological observation data using established meteorological assessment indicators for the occurrence of wheat freeze injury [39, 40]. Meteorological observation stations, however, are usually located in the suburbs of cities far away from the farmland, and the meteorological data they acquire is unlikely to accurately reflect the microclimatic conditions existing at actual wheat production facilities; for this reason, the results produced by wheat freeze injury severity monitoring methods using meteorological indicators often exhibit significant errors. A potentially more reliable approach to wheat freeze injury severity monitoring might be to make use of the fact that wheat freeze injury is associated with a significant color change of the leaves, stalks, and other organs of affected plants. The study presented in this paper proposes a method for monitoring wheat freeze injury severity utilizing high-definition digital images of wheat canopies acquired in situ at wheat fields throughout Shandong Province. It investigates the correlations between the wheat freeze injury severity grade, a number of meteorological factors and a number of parameters extracted from the leaf color distribution in wheat canopy images.

Our results indicate that the sensitivity of the response of both the meteorological factors we investigated and the CGSD parameters we extracted from the leaf color distribution to the freeze injury severity inflicted upon the canopy was sufficient to merit using them as indicators. However, in terms of accuracy, the methods using the leaf color as input consistently outperformed those using meteorological factors as inputs. A possible explanation for this finding is that the wheat freeze injury severity is not determined by a single factor: in addition to meteorological factors, the growth stage of the affected wheat population also plays an essential role in the severity of the impact of cold temperatures, and because the leaf color directly depends on the plant growth stage, freeze injury severity assessment methods based on leaf color implicitly include the growth stage as a factor. In terms of accuracy, we found that leaf-color-based wheat freeze injury severity classification methods deploying algorithms that do not involve the use of prior data (notably K-means clustering and systematic clustering) typically exhibited an accuracy of less than 75%, whereas the accuracy of Bayesian classification methods, which do involve the use of prior data, turns out to be better. When applying the Bayesian leaf-color-based wheat freeze injury severity classification we developed for this study to the images acquired at the experimental stations in Gaomi and Jiang sites, we found that the average accuracy exceeded 85%, which meets commercial requirements.

With respect to the results obtained at the experimental station in Qihe, however, the accuracy of our Bayesian model was only 66.13%, which was likely due to the fact that the station encompasses several different ecological zones. In an attempt to improve the accuracy of our model in this kind of situation, we took a closer look at the CGSD parameters. It has been suggested that, although the CGSD parameters can adequately characterize the leaf color information needed to assess the wheat freeze injury severity, a degree of covariance exists among these parameters [31]. This covariance amplifies the contributions of specific parameters, which in turn affects the determination accuracy of classification models using CGSD parameters as inputs. Zhang et al. (2020) [41] proposed the use of factor analysis, principal component extraction and other methods to construct new composite factors from more than one hundred meteorological factors. After the development of a new zoning index, those new factors could replace the traditional factors as inputs for classification models, thereby improving their accuracy by solving the problems caused by using too many input variables [41]. It should be noted that composite factors have certain limitations: they can be difficult to calculate and extract and they tend to require complication transformations before they can be applied by a model, which might constrain the large-scale application of models using them. For our study, however, these limitations were surmountable, so in order to improve the accuracy of our Bayesian model, we carried out a factor analysis and used the result to optimize the model by limiting the factors used as inputs to the Bayesian model to two composite factors calculated from the six most independent key parameters, thereby removing any inaccuracies caused by covariance between the CGSD parameters. The six key parameters we identified were *R*_Kurtosis_, *G*_Skewness_, *G*_Median_, *G*_Mode_, *Y*_Skewness_, and *G*_Mean_. The accuracy of the optimized classification model reached 90.32% when applied to the data acquired at Qihe station, while the accuracy when applied to the acquired at Gaomi and Jiyang station was similar to that of the model before optimization.

Because the leaf color parameters used in our study were based on descriptive statistics associated with a skewed color distribution, the number of basic leaf color parameters that the traditional RGB color model could extract was greatly expanded. However, the classification model could only maintain a high accuracy in the presence of precise or high correlation between the parameters. Our results confirm that factor analysis can be used to determine the key leaf color parameters and remove inaccuracies caused by amplification of certain parameters due to factor covariance, thus providing a promising approach to constructing a scientifically sound and stable Bayesian wheat freeze injury severity classification model that is accurate enough to meet the requirements for commercial wheat freeze severity monitoring.

## 5. Conclusions

This paper explores the potential for utilizing the color information in digital images of crop canopies for classifying wheat freeze injury classes. Our results lead to the following conclusions:

1. Both meteorological factors and the color information in digital images of wheat canopies correlate well with the observed wheat leaf freeze injury severity, which proves the concept of using meteorological factors and leaf color information to classify wheat freeze injury severity.
2. The accuracy of models using leaf color information as input was significantly higher than that of models using meteorological factors as inputs, whether they involved the use of prior data or not.
3. At the experimental stations in Gaomi and Jiyang, the classification accuracy of the Bayesian classification model we tested, which involved the use of prior data in the form of manually assessed wheat freeze injury severity classifications, was over 85%, which was significantly higher than that of the systematic clustering and K-means clustering models that did not involve the use of prior data. At the experimental station in Qihe, however, which encompassed different ecological zones, the accuracy reached only 66.13%, but after optimizing our Bayesian wheat freeze injury severity classification model through factor analysis, its accuracy was over 90%, which is significantly better than before optimization and suggests that the optimized model might be applicable to a wide range of wheat production sites.

The identification method of wheat freeze injury severity obtained in this paper is based on the rule that wheat canopy color characteristics are different under different freeze injury severity. This method can also be applied to the intelligent judgment of other agrometeorological disasters such as drought, high temperature, etc. However, we found that the collected wheat canopy image needed to be eliminated in the research due to light interference and lens attachment interference, which greatly reduced the utilization rate of the images and the efficiency of analysis and application. In the future, we should explore ways to solve this problem. On the one hand, the equipment can be optimized from the crop image acquisition terminal; In addition, correction algorithms for canopy images under different light conditions can also be explored.
